# Bacterial and Viral Coinfections with the Human Respiratory Syncytial Virus

**DOI:** 10.3390/microorganisms9061293

**Published:** 2021-06-13

**Authors:** Gaspar A. Pacheco, Nicolás M. S. Gálvez, Jorge A. Soto, Catalina A. Andrade, Alexis M. Kalergis

**Affiliations:** 1 Departamento de Genética Molecular y Microbiología, Facultad de Ciencias Biológicas, Millennium Institute of Immunology and Immunotherapy, Pontificia Universidad Católica de Chile, Santiago 8320000, Chile; grpacheco@uc.cl (G.A.P.); nrgalvez@uc.cl (N.M.S.G.); jasoto6@uc.cl (J.A.S.); cnandrade@uc.cl (C.A.A.); 2 Departamento de Endocrinología, Facultad de Medicina, Pontificia Universidad Católica de Chile, Santiago 8320000, Chile

**Keywords:** hRSV, coinfection, IAV, hMPV, HPIV, hRV, *S. aureus*, *P. aeruginosa*, *S. pneumoniae*, respiratory infections

## Abstract

The human respiratory syncytial virus (hRSV) is one of the leading causes of acute lower respiratory tract infections in children under five years old. Notably, hRSV infections can give way to pneumonia and predispose to other respiratory complications later in life, such as asthma. Even though the social and economic burden associated with hRSV infections is tremendous, there are no approved vaccines to date to prevent the disease caused by this pathogen. Recently, coinfections and superinfections have turned into an active field of study, and interactions between many viral and bacterial pathogens have been studied. hRSV is not an exception since polymicrobial infections involving this virus are common, especially when illness has evolved into pneumonia. Here, we review the epidemiology and recent findings regarding the main polymicrobial infections involving hRSV and several prevalent bacterial and viral respiratory pathogens, such as *Staphylococcus aureus*, *Pseudomonas aeruginosa*, *Streptococcus pneumoniae*, *Haemophilus influenzae*, *Moraxella catarrhalis*, *Klebsiella pneumoniae*, human rhinoviruses, influenza A virus, human metapneumovirus, and human parainfluenza viruses. As reports of most polymicrobial infections involving hRSV lack a molecular basis explaining the interaction between hRSV and these pathogens, we believe this review article can serve as a starting point to interesting and very much needed research in this area.

## 1. Introduction

### hRSV Epidemiology and Structure

The human respiratory syncytial virus (hRSV), currently known as human orthopneumovirus, is a member of the *Orthopenumovirus* genus from the *Pneumoviridae* family and is the principal etiological agent responsible for causing acute lower respiratory tract infections (ALRTIs) in infants, immunocompromised patients, and the elderly [1,2]. Infection by hRSV can cause mild symptoms, such as fever, coughing, wheezing, and even severe symptoms, including bronchiolitis and pneumonia, leading to hospitalization [3]. It has been estimated that hRSV infections cause 3.2 million hospitalizations per year, out of which 1.4 million hospital admissions correspond to infants under six months old [4]. Curiously, hRSV infections were the most frequent respiratory infection among infants during 2020 in Nashville, Tennessee, USA, causing an even higher number of hospitalizations than other viral infections, despite the current SARS-CoV-2 pandemic [5]. Up to date, there are no approved vaccines to prevent hRSV disease. However, there is a palliative treatment based on a monoclonal antibody (Palivizumab) used mainly in high-risk populations, such as preterm infants and children with respiratory or cardiac comorbidities [6,7].

The structure of hRSV consists of a pleomorphic viral particle that contains a single-stranded, non-segmented, negative-sensed viral RNA of 15 kb of length [8]. This genome comprises ten genes that encode nine structural proteins and two non-structural (NS) proteins [9]. Structural proteins can be further classified into surface proteins and internal proteins, depending on the position of the protein relative to the surface of the virion. Firstly, three proteins can be identified on the surface of hRSV: the fusion protein (F), the attachment glycoprotein (G), and the small hydrophobic protein (SH). Both G and F proteins are responsible for the attachment and fusion of the virus with the host cell [10]. The SH protein is not essential for the infection of the host, but it has been reported that it can prevent the activation of the TNF-signaling pathway, and it can also act as a viroporin [11,12,13]. The ribonucleoprotein (RNP) complex can be found on the interior of hRSV and is composed of the nucleoprotein (N), the phosphoprotein (P), the large protein/RNA-dependent RNA polymerase (L), and the viral RNA. The RNP complex can protect the RNA genome from chemical and physical damage and provide the essential replication and transcription machinery [14]. The genome of hRSV codes for two M2 proteins (M2.1 and M2.2), as the gene responsible for encoding this protein exhibits two open reading frames (ORF). These proteins have been reported to play a role in the transcription and replication of the viral RNA [15,16]. There is a physical association between the RNP complex, M2.1, and the matrix protein (M) [14]. Finally, NS1 and NS2 proteins play a role during the immune response evasion since these proteins prevent the secretion of type I interferons (IFN-I) [17].

Initial infection with hRSV can coincide with a viral or bacterial infection, causing what is known as a coinfection [18]. hRSV infection can increase the risk of ALRTI in infants when there is coinfection with viruses or bacteria [19,20]. However, in the case of viral coinfections with hRSV, the risk of developing ALRTI does not increase with every virus, as only a few of them—such as human rhinovirus, parainfluenza virus 3, and human metapneumovirus—may cause this [19]. For bacterial coinfections with hRSV, mechanical ventilation was required for a more extended time compared to infants that were only infected with hRSV [20]. Superinfections can also occur during hRSV infections, a phenomenon characterized by the infection with one pathogen after initial infection with another pathogen [21]. Superinfections can occur due to the damage of the epithelium or the evasion of the immune response caused by the first pathogen, worsening the symptoms in the case of hRSV [22]. This review will discuss the different types of co- and superinfections reported in association with hRSV and the possible mechanisms that make these plausible.

## 2. hRSV Coinfections with Pathogenic Respiratory Bacteria

Viral and bacterial coinfections have recently received much attention since they are commonly linked to community-acquired pneumonia (CAP). Coinfections can increase the severity of the disease and even increase mortality rates [23]. In general, the rate of coinfection in respiratory diseases can reach up to 68% of the hospitalized patients [24]. Coinfections could be explained by a possible dysregulation of the host immune response upon infection with one pathogen, rendering the posterior infection with the other pathogen more accessible [25].

**Figure 1:**
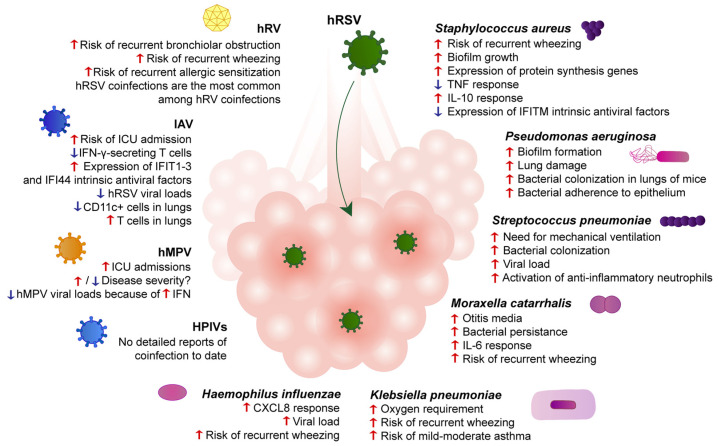
hRSV coinfections with bacteria and viruses modulate lung pathogenesis and clinical outcomes. Bronchi are depicted during infection with hRSV. Descriptions beneath the bolded name of each pathogen represent differences between the coinfection between hRSV and the respective pathogen and hRSV single-infection. Red arrows represent an upregulation or more marked response. Blue arrows represent a downregulation or diminished response.

The most common coinfections between hRSV and bacteria involve *Staphylococcus aureus*, *Pseudomonas aeruginosa*, *Streptococcus pneumoniae*, *Moraxella catarrhalis*, *Haemophilus influenzae*, or *Klebsiella pneumoniae* [24]. It was reported that hRSV–virus coinfection does not increase the severity of the disease, but that hRSV–bacteria coinfection increases disease severity in infants [26]. Patients infected with hRSV tend to register more coinfections with bacteria than any other viral pathogen [27]. Moreover, infants hospitalized due to hRSV-caused bronchiolitis usually also present respiratory tract bacterial infections [28]. Unfortunately, even though hRSV–bacteria coinfections pose a severe threat to infants, there is not much information regarding this subject. Moreover, while most reports gather extensive epidemiological data, experimental setups in vitro and in vivo are scarce. Figure 1 summarizes our current understanding of the main differences between hRSV–bacteria coinfections and the corresponding mono-infections.

### 2.1. Staphylococcus aureus

*Staphylococcus aureus* is a Gram-positive commensal and opportunistic bacterium, which is one of the leading bacterial pathogens causing community-acquired pneumonia [29,30,31,32]. Several antibiotic resistance genes and cassettes were discovered among *S. aureus* strains, and methicillin-resistant *S. aureus* (MRSA) represents a significant social and economic burden because of its high incidence and scarce treatment alternatives [33,34,35,36].

hRSV and *S. aureus* coinfection is among the most prevalent hRSV–bacteria respiratory coinfections. Several studies have reported numerous cases of coinfection with *S. aureus* in nasal swabs of children under five years old with pneumonia [37,38,39]. Coinfection has been associated with a higher probability of developing pneumonia than hRSV infection alone, as well as with a higher probability of having abnormal chest X rays, requiring oxygen supplementation, shortness of breath, wheezing, fever, higher counts of white blood cells and platelets, and higher C reactive protein (CRP) in serum [40]. Importantly, the risk of recurrent wheezing after hRSV infection has been shown to increase dramatically when children under three years old suffer from an hRSV–*S. aureus* coinfection [40]. Moreover, there have been rare reports of necrotizing pneumonia during MRSA infection and hRSV–MRSA coinfection [41,42].

Viral and bacterial respiratory coinfections are especially critical for cystic fibrosis (CF) patients, who commonly present bacterial and viral infections by *Staphylococcus aureus*, *Pseudomonas aeruginosa*, human rhinoviruses, hRSV, and influenza viruses [43]. Interestingly, it has been shown that infection with hRSV promotes *S. aureus* biofilm growth in an in vitro CF airway epithelial cell (AEC) model without altering the attachment of *S. aureus* to the AECs [44]. The proliferation of *S. aureus* in CF AECs infected with hRSV increased in three out of five clinical isolates obtained from sinonasal swabs from CF patients, indicating that hRSV promotes *S. aureus* growth in CF mucosal tissues [44]. Moreover, RNA-seq analyses showed that genes involved in protein translation, ribosome biosynthesis, and amino acid and lipid metabolism were highly upregulated in coinfected AECs compared to AECs infected with *S. aureus* alone [44]. Further analyses showed that the highly pro-inflammatory TNF response elicited by *S. aureus* was downregulated during coinfection with hRSV. The immunoregulating cytokine IL-10 was upregulated in coinfected AECs when compared to *S. aureus*-infected AECs [44]. Higher levels of IL-10 were associated with *S. aureus* persistence [45], which could explain why *S. aureus* coinfections are so common in hRSV-caused pneumonia. On the other hand, coinfected AECs showed downregulated expression of intrinsic antiviral factors of the interferon-inducible transmembrane (IFITM) family, which could promote hRSV infection [44]. Moreover, coinfected AECs exhibited a different pattern of expression of mucin genes [44].

Even though these experiments were performed in a CF AEC model, they could shed light on the interactions between hRSV and *S. aureus* during coinfection in healthy patients. It appears that coinfection with both pathogens results in a change in cellular metabolism and an imbalanced and inappropriate immune response against either pathogen. Thus, it would be interesting to evaluate changes in the immune response elicited by the coinfection of these pathogens in an animal model to further understand the details of the response against both pathogens and develop better treatments for hRSV–*S. aureus* pneumonia. Moreover, it would be interesting to evaluate whether these phenomena also occur in healthy AECs.

Since CF patients are particularly susceptible to bacterial and viral respiratory tract infections, prophylactic approaches to some of these infections are of great interest. Palivizumab, a humanized monoclonal antibody, is the only approved preventive approach against hRSV [3]. Even though this antibody effectively prevents hRSV infection in healthy children, its efficacy in CF infants remains controversial [46,47,48,49]. Moreover, it does not control the eventual infection of essential pathogenic bacteria in CF patients, such as *S. aureus* or *Pseudomonas aeruginosa*, nor reduces the amount of medical attention these patients need [46]. Interestingly, it has been shown that the use of Palivizumab during infancy is associated with long-term changes in the microbiota composition of preterm infants, favoring the growth of pathogenic taxa of bacteria [50]. Given that hRSV infection of epithelial cells could potentially enhance *S. aureus* growth in the airways, it would be interesting to investigate further whether Palivizumab can indirectly protect against bacterial infections of the respiratory tract of healthy infants.

Even though *S. aureus* and hRSV coinfections are so common, there is a lack of information regarding the possible molecular and cellular mechanisms that favor them in healthy individuals. While most studies focus on CF patients [44,45], virtually no reports study interactions between the host, hRSV, and *S. aureus* in non-CF patients. Indeed, valuable information could be extracted from this kind of study, contributing to our understanding of polymicrobial hRSV infections.

### 2.2. Pseudomonas aeruginosa

Another typical bacterial pathogen found during hRSV–bacteria coinfections is *Pseudomonas aeruginosa*, an opportunistic pathogen of high prevalence in CF and immunocompromised and mechanically ventilated patients [51].

By implementing a co-culture model to study biofilm formation in the respiratory epithelium, it was shown that a primary hRSV infection promotes biofilms by *P. aeruginosa*. The mechanism associated with this phenomenon was attributed to the antiviral immunity triggered by the natural hRSV infection, mediated by the IFN pathway in the respiratory epithelium. This response produces changes in the iron homeostasis of the apical airway epithelium, increasing the availability of this metal, which is essential for forming *P. aeruginosa* biofilms in either abiotic or biotic environments [52].

Additionally, the effect of hRSV infection on the adherence of *P. aeruginosa* was also evaluated in various cell lines, such as HEp-2, IB3-1, and A549 [53]. It was shown that hRSV infection modulates the adherence of *P. aeruginosa* to these epithelial cells. This adherence enhancement is both dose- and time-dependent for each cellular type. One possible mechanism that could explain this phenomenon is that the bacterium could bind surface proteins expressed on epithelial cells during an hRSV infection or bind to viral glycoproteins found on the surface of infected cells [53].

Since coinfection between hRSV and *P. aeruginosa* produces a chronic infection in CF patients, many studies have focused on coinfections in CF models [54]. A study evaluated changes in volatile metabolites produced by the bacterial infection by *P. aeruginosa*, viral infection by hRSV, and the coinfection between both in an in vitro CF bronchial epithelial model (CFBE). Here, the authors found significant differences between some volatile components modulated during infection with hRSV in the presence or absence of *P. aeruginosa* in CFBE. However, no apparent differences in the signature of volatile components were observed between CFBE infected with *P. aeruginosa* alone or *P. aeruginosa*–hRSV coinfection, which suggests that the composition of volatile components is dominated by *P. aeruginosa,* rather than hRSV infection, during coinfection [54].

Given the significant impact generated by a coinfection that presents *P. aeruginosa* in CF patients and its capacity to promote antibiotic resistance, the use of novel therapies is necessary. An engineered cationic antimicrobial peptide (eCAP) has been developed and evaluated on the formation of *P. aeruginosa* biofilms. It was concluded that WLBU2, an eCAP, disrupted the formation of *P. aeruginosa* biofilms without generating toxic effects for the pulmonary epithelium while also reducing bacterial viability [55]. Moreover, this peptide was able to mitigate hRSV infectivity, suggesting that it could be a good candidate not only against these pathogens but also for others considered among the ESKAPE group (*Enterococcus faecium, Staphylococcus aureus, Klebsiella pneumoniae, Acinetobacter baumannii, P. aeruginosa,* and *Enterobacter*) [55].

Some in vivo studies have shown that a simultaneous hRSV and *P. aeruginosa* infection leads to an increase in the number of bacterial CFU in the lungs of mice. Moreover, coinfection resulted in enhanced lung damage, characterized by decreased respiratory capacity compared to animals infected only with *P. aeruginosa* [51]. However, the most important limitation of this study was the evaluation only 24 h later of the simultaneous virus–bacteria infection.

### 2.3. Streptococcus pneumoniae

*Streptococcus pneumoniae* is another major pathogen responsible for hRSV–bacteria coinfections. A study performed in 2006 correlates the incidence of bacterial coinfections in children with a severe hRSV infection admitted in the pediatric insensitive care unit with a more prolonged need for mechanical ventilation support. Here, the authors found that among the 165 children evaluated in the study, 98 of them presented bacterial colonization, with *Haemophilus influenzae* being the most prevalent, followed by *S. aureus*, *Moraxella catarrhalis,* and *S. pneumoniae* [20]. Interestingly, these coinfections have also been identified in elderly patients. As a matter of fact, 42.3%–79.7% of hRSV infections in the elderly are accompanied by bacterial pneumonia [56,57]. A recent study found that *S. pneumoniae* was present in 12.1% of hRSV-infected elderly patients and represented the most common bacterial pathogen found among hRSV–bacteria coinfections [57].

A recent retrospective study performed in Australia analyzed nasal swab samples from children under two years old. A total of 54 cases of hRSV infections were registered among 47 children, and the predominant coinfections found were associated with *S.* *pneumoniae* (61.1%), followed by *Moraxella catarrhalis* (48.1%) and *H. influenzae* (14.8%) [58]. Interestingly, some events of hRSV, *S.* *pneumoniae,* and *M. catarrhalis* coinfections were detected before or after an hRSV infection. However, none of these events could be associated with an enhanced predisposition to hRSV infection because of previous infection events with either bacteria [58].

Although it is currently known that coinfections and superinfections between hRSV and specific taxa of bacteria are prevalent, a mechanism that explains their prevalence or symptomatic differences when comparing to single infections is unknown. Some reports have shown that an initial infection with hRSV promotes an increase in the bacterial colonization in the nasopharyngeal tissue when *S. pneumoniae* is found in an opaque phase, while decreasing the adherence capacity of the bacteria during the transparent state [59]. In contrast, when *S. pneumoniae* infects the respiratory tract first, subsequent hRSV superinfection has been observed to increase the capacity of infection of hRSV in an in vitro model using well-differentiated normal human bronchial epithelial cells. Interestingly, this effect was shown to be dependent on the specific *S. pneumoniae* serotype [60]. Experiments using an in vivo model using a primary *S. pneumoniae* infection three days before an hRSV infection showed an increase in the viral load in nasal lavage compared to hRSV mono-infected mice. Similar results were also found in a cotton rat model, in which an initial *S. pneumoniae* infection promoted an increase in the hRSV viral load in nasal washes [60].

On the other hand, immunological reports in humans have shown that coinfection between hRSV and bacteria promotes the activation of a subset of neutrophils with anti-inflammatory capacities [61]. This type of cell could potentially generate a suppression of T cells and thus help a viral infection. However, this hypothesis is yet to be proved [62].

### 2.4. Other Pathogenic Respiratory Bacteria

Other opportunistic bacteria associated with hRSV illness and pneumonia are *Klebsiella pneumoniae*, *Moraxella catarrhalis*, and *Haemophilus influenzae*.

One study evaluated whether an hRSV infection can increase *Moraxella* proliferation in an otitis media model in chinchillas. It was found that the infection of chinchillas with *M. catarrhalis* two days before hRSV infection promotes the ascent of *M. catarrhalis* from the upper respiratory tract to the middle ear in these animals, facilitating otitis media [63]. Interestingly, 100% of the chinchillas presented nasopharyngeal colonization of *M. catarrhalis* 7 days post-hRSV infection. Surprisingly, when the use of a non-typeable *Haemophilus influenzae* (NTHI) was included four days prior to the initial infection scheme with *M. catarrhalis* and hRSV, *Moraxella* was found up to 17 days post-hRSV infection, suggesting that the coinfection of NTHI and hRSV enhances the colonization and infection of *M. catarrhalis* in the chinchillas even more than hRSV alone [63].

In this line, a recent study in Beijing monitored hRSV-infected children under six months of age until they reached three years of age. Here, the authors identified an increase in sibilance development in the children that exhibited coinfections with *Haemophilus* or *Moraxella*, which was determined using 16S rRNA-based sequencing. Moreover, coinfection was correlated with an increased concentration of LPS and some cytokines and chemokines in the airways. Remarkably, coinfection with *Moraxella* was associated with IL-6 secretion while *Haemophilus* was associated with CXCL8 [64], both of which are detrimental to hRSV pathology resolution. A recent report studied children under six months of age infected (or not) with hRSV to evaluate changes in nasopharyngeal microbiota composition.

Interestingly, the microbiota of hRSV-infected children shifted to favor an over-colonization of two opportunistic pathogens: *Haemophilus* and *Achromobacter* [65]. Similar to previous findings, the over-colonization by *Haemophilus* was correlated with an increase in the amount of CXCL8 in the mucosal tissues and an increase in the viral load of hRSV [64,65]. Interestingly, viral replication decreased when treating human bronchial epithelial cells (16HBE14o-) with commensal NTHI prior to hRSV infection [66]. This effect was not observed during infection with the influenza virus, suggesting a specific interaction between NTHI and hRSV [66].

*K. pneumoniae* is another bacterial pathogen commonly found in hRSV-positive samples, even though statistics vary among locations. While one study detected the presence of this bacterium in 66% of hRSV cases and in 58% of hRSV-caused pneumonias [67], another reports lower rates for coinfection with *Klebsiella*, reaching only 10% of co-detection of total hRSV detection [40]. hRSV–*Klebsiella* coinfections have been associated with higher oxygen requirement and higher C reactive protein than hRSV mono-infections and a higher risk of developing recurrent wheezing in children under three years old [40]. Other studies have also found and correlated the presence of *K. pneumoniae, M. catarrhalis,* and *H. influenzae* in hRSV-positive samples with a higher risk of developing recurrent wheezing [64], and correlating *K. pneumoniae* colonization with the development of mild to moderate asthma [68]. One possible explanation for this is that bacteria can colonize the respiratory tract more efficiently during an hRSV infection since the virus promotes Th2 polarization of T cells [69], diminishing the Th17 polarization needed for neutrophil recruitment and bacteria elimination in the airways [70]. However, one study found that the induction of a Th2 response in a model of allergic sensitization had no impact on *K. pneumoniae* burden [71]. Studies evaluating how and why hRSV infections could promote *Klebsiella* infections are most certainly needed to understand why these coinfections are so common.

## 3. hRSV Coinfections with Other Respiratory Viruses

In this section, the coinfections with hRSV and other respiratory viruses will be discussed. Although coinfections with hRSV are reported for several respiratory viruses, such as human rhinoviruses, influenza A virus, human metapneumovirus, parainfluenza virus, bocaviruses, adenoviruses, and coronaviruses, we will focus only on the first four since they are the major cause of ALRTI in infants [72,73,74]. Figure 1 summarizes key points associated with hRSV–virus coinfections. It is important to note that the sanitary measures adopted worldwide in the year 2019 and onwards to stop spreading the ongoing pandemic of SARS-CoV-2 (such as social distancing, wearing a facemask, and recurrent handwashing) have had significant repercussions on the circulation of other respiratory viruses [75]. Therefore, reports of SARS-CoV-2 and hRSV coinfection are not available to date.

### 3.1. Human Rhinovirus

Human rhinoviruses (hRVs) were first described in 1950 and are usually associated with the common cold [76,77,78,79]. Although primarily responsible for mild upper respiratory tract infections in children and vulnerable populations, hRVs can cause severe lower respiratory tract infections (LRTIs) [78,80]. To date, no approved vaccines are available against hRVs, as over 100 serotypes with high antigenic variability constantly circulate [77,78]. hRVs are members of the *Picornaviridae* family and the *Enterovirus* genus [76,77,78]. The genome of hRVs consists of a positive-sensed, single-stranded RNA molecule, about 7.2 kb in size. Its genome codes for a single gene which, upon transcription and translation of a single polypeptide, is cleaved for the generation of four structural and seven non-structural proteins [76,77,78]. Since no animal models are currently available for the study of hRVs, most of the literature on coinfection with hRSV focuses on epidemiological reports.

Studies have shown that other respiratory viruses are not frequently detected in samples from children infected with hRVs, suggesting that this virus might have mechanisms to impair the infection capacity of different respiratory viruses [81,82]. Despite this, coinfections are still detected and reported throughout the literature, and most of these are associated with hRSV [19,79,81,83,84,85,86,87]. Accordingly, severe cases of hRV disease seem to be related to hRSV coinfections [19,79,88]. Remarkably, it has been shown that hRSV–hRV coinfections predispose children to recurrent bronchiolar obstruction, wheezing, and allergic sensitization [80]. This predisposition mechanism is currently unknown. However, atopic conditions (defined as allergic rhinitis or atopic dermatitis) or asthmatic family history were also linked to this phenomenon, so there is probably a genetic component working in this regard [80].

Interestingly, unlike what is seen for other respiratory viruses such as hRSV and IAV, hRV does not induce a significant change or destruction of the infected cells in the airway epithelium [89,90]. This phenomenon may have considerable relevance during superinfections, as viruses such as hRSV might still be able to induce the shedding and destruction of infected cells, disrupting the natural infective cycle of hRVs. Therefore, the temporal sequence of infection is relevant when performing superinfection and even coinfections studies in these viruses, as reported before [91].

### 3.2. Influenza A Virus

Influenza viruses, particularly influenza A virus (IAV), are responsible for seasonal flu, affecting both adults and vulnerable populations [92,93,94]. The economic and social burden associated with this virus is outstanding, with seasonal flu causing 250,000–650,000 deaths every year worldwide [92,93,94]. IAV is always on the spotlight as a possible source for pandemic outbreaks [92,94]. Remarkably, most respiratory diseases are termed influenza-like, as this pathogen causes most of the hallmark symptoms associated with acute respiratory tract infections (ARTIs), such as sore throat, coughing, fever, muscle pain, and fatigue [92,93,94,95]. Vaccines approved against this pathogen consist of either live attenuated virus, inactivated virus, or recombinant proteins. They usually induce protection against three or four different strains and promote sterilizing immunity [92,95]. However, since antigenic variations are expected for IAV, vaccine composition and vaccination effectiveness vary from year to year [92,95]. Particularly, IAV belongs to the *Orthomyxoviridae* family and the *Alphainfluenzavirus* genus [93,96]. The genome of IAV is a negative sensed, single-stranded RNA divided into eight segments, each with its promotors and coding regions, reaching a total size of 13.5 kb [93]. The segmentation of its RNA and the zoonotic capacities increase its mutation rates and contribute to genetic variability [92,93,94,95].

Since there is a close temporality in the circulation of influenza viruses and hRSV (overlapping in some years), detecting coinfections between these viruses is common [72,73,75,97,98,99,100,101,102,103]. Although hRV coinfections are more frequent for A(H1N1) pandemic IAV, hRSV is the most common virus detected in coinfections for seasonal IAV, which could be related to viral circulation temporality (i.e., year-long for hRV and winter season for hRSV) [98,99]. Overall, IAV–hRSV coinfections in children and adults result in more severe diseases, with an increased risk of admission to the ICU or even death [98,100]. This increase in the severity of the disease could be related to decreased percentages of IFN-γ-secreting T cells reported during IAV–hRSV coinfections [101]. Despite this, IAV–hRSV coinfections detected in surveillance studies are usually less than predicted given their prevalence, with studies showing that actual cases of coinfection are up to six times less than expected [102].

The use of animal models and cell lines has shed some light on specific mechanisms underlying IAV–hRSV coinfections and superinfections [104]. In these lines, recent reports have shown that IAV infection inhibits hRSV in vitro, in superinfection models of HEp-2 cells independently of which virus infects first [104]. A typical antiviral response induces the secretion of type I, II, and III interferons (IFN-I, -II, -III, respectively) and the subsequent activation of interferon-stimulating genes (ISGs) pathways [104,105,106]. These pathways also lead to the interferon-induced protein expression with the tetratricopeptide (IFIT) proteins family [104,105,106]. Particularly, the complex IFIT-1-3 has been shown to impair the translation of viral mRNA [104,107]. Interestingly, IFIT1-3 was shown to play a significant role in this inhibition of hRSV infection [104]. This protein complex was upregulated during IAV infections. Moreover, the silencing and overexpression of IFIT1-3 resulted in increased and reduced hRSV viral loads, respectively [104]. The same was described for IFI44, another ISG induced during IAV infection [104]. Therefore, the induction of IFIT1-3 and IFI44 by IAV infection modulates coinfections and superinfections with hRSV. Coinfection and superinfection of BALB/c mice with IAV and hRSV lead to similar yet not equal results [108]. Superinfection models, independently of which virus was added first, had no impact on the replicating capacities of either virus [108]. However, it did impact the expression of hRSV genes in different ways [108]. Remarkably, IAV–hRSV coinfection led to reduced *f-, g-,* and *ns1-hrsv* gene expression, which is in line with cell lines studies [104,108]. Coinfections and superinfections also reduced numbers of CD11c^+^ cells in the lungs at early time points and increased CD3^+^ NK1.1^+^, CD3^+^ CD4^+^, and CD3^+^ CD8^+^ cells [108]. Although these data must be considered cautiously (i.e., BABL/c mice do not express canonical NK1.1 [109]), there seems to be a correlation between in vivo and in vitro reports, suggesting that IAV impairs the infective capacity of hRSV during coinfections and superinfections, which could be in line with the lower-than-expected detection rates of IAV–hRSV coinfections reported in surveillance studies [102].

### 3.3. Human Metapneumovirus

Human metapneumovirus (hMPV) is among the leading respiratory viruses responsible for hospitalizations of children and vulnerable populations worldwide [8,110]. This virus was first described in 2001 and is primarily accountable for ARTI and LRTI cases [8,110,111]. hMPV belongs to the *Pneumoviridae* family and the *Metapneumovirus* genus [8,110,112], and its genome is a negative sensed, single-stranded RNA molecule of about 13.3 kb in size [8,110]. This virus exhibits remarkable similarities with hRSV, both in its molecular structure and the disease symptoms it elicits [8,110]. To date, no licensed vaccines are available against this virus, although several preclinical prototypes are being tested [113].

Several reports are available describing hRSV–hMPV coinfections, with mixed conclusions [114,115,116]. A recent meta-analysis showed that coinfections with these viruses lead to a higher risk of ICU admission and more extended hospital stay than coinfections with other viruses [114]. However, no changes in the use of supplemental oxygen, mechanical ventilation, or reported deaths were found among the evaluated studies [114]. Studies have also shown that clinical manifestations during hRSV–hMPV coinfections seem to remain invariable compared to mono-infection [115]. These differences among studies may be related to the age range evaluated in each one, as children under 6 months old seem to be more vulnerable to increased disease severity than older children [114,115,116,117]. Accordingly, mixed reports indicate that hRSV–hMPV coinfections are [118,119] and are not [120] detected in children with severe ARTI cases. However, cohort sizes among these studies have significant impacts, as bigger cohorts indicate that coinfections are detected [118,119]. Therefore, despite not being significantly frequent, hRSV–hMPV coinfections exhibit more severe disease symptoms than mono-infections.

Using three-dimensional cell cultures, a model for the study of airway epithelium, some mechanisms underlying hRSV–hMPV coinfections have been elucidated [121]. As also seen for other respiratory viruses, the presence of hRSV in the culture partially inhibited the replicative capacities of hMPV in both coinfection and superinfection models [121]. This effect was not seen for hRSV, as viral loads remained similar independently of the presence of hMPV in culture [121]. This impairment seems to be directly related to the expression of IFN-I and IFN-III, as the external addition of these molecules or knock-out (KO) of signaling pathways associated with IFN expression (such as STAT1 or IL28RA) decreased and enhanced the replicating capacities of hMPV, respectively [121]. Interestingly, these modifications did not have a significant impact on hRSV [121]. Since IFN expression is a common trait during viral infections, this could explain the impaired replicating capacities of hMPV in coinfections and superinfections with hRSV [121]. This phenomenon could also explain why hRSV–hMPV coinfection surveillance reports show mixed conclusions.

### 3.4. Parainfluenza Viruses

Human parainfluenza viruses (HPIVs) are one of the major etiological agents responsible for ALRTIs. HPIVs are a polyphyletic taxonomic group belonging to the *Paramyxoviridae* family, are closely related to influenza viruses, and possess a negative-sensed, single-stranded, non-segmented RNA genome around 15 kb in length [122]. There are four major serotypes of HPIVs, named HPIV1, HPIV2, HPIV3, and HPIV4 [122,123]. While HPIV1 and HPIV3 belong to the *Respirovirus* genus, HPIV2 and HPIV4 belong to the *Orthorubulavirus* genus [122]. Although epidemiological data support their co-circulation with hRSV [124], there are no reports of enhanced illness due to HPIV–hRSV coinfection. Furthermore, reports on virus–virus coinfection evaluating the presence of HPIV genetic material do not often detail the identity of which viruses tend to coinfect with which other viruses [74,125]. Moreover, we have not found any evidence or studies assessing any possible interactions between these viruses, neither in in vitro nor in in vivo models.

However, one current vaccine candidate against hRSV makes use of a strain of bovine parainfluenza virus expressing the F protein of hRSV and induces protective immunity in both hamsters and Rhesus monkeys [126]. Although this approach takes advantage of host restriction by using a bovine PIV strain, it could also tell us that potential underlying HPIV–hRSV interactions could be of biological and medical interest. The existing similarities between both viruses could potentially modulate the immune response elicited to either or both of them during coinfections or superinfections. We would expect coinfection rates to be lower than expected, as seen for IAV–hRSV coinfections, because of similarities between IAV and HPIVs. However, any potential effects of exacerbation or amelioration of disease because of coinfection, compared to hRSV or HPIV infection alone, remain to be determined. If such an interaction exists, it would be interesting to determine which components of the viruses or the host’s immune response, such as similarities between surface proteins or fine-tuning of IFN responses, respectively, contribute to exacerbation or amelioration of disease.

## 4. A Summary of Molecular and Cellular Mechanisms Pertaining to hRSV Coinfections

Despite the prevalence and severity of hRSV coinfections, both with pathogenic bacteria and viruses, studies aiming to dissect molecular and cellular mechanisms during coinfections are scarce. Thus, we believe that further research is required in this respect since it will help to better understand and provide a molecular basis for the observed epidemiological data. This section summarizes the few articles that provide cellular and/or molecular data of hRSV coinfections.

In general, it has been shown that hRSV coinfection with bacteria tends to favor both bacterial and viral replication in the lungs [44,52,60,65]. While increased viral replication could be explained by the downregulation of intrinsic antiviral factors in the host cell [44], enhanced biofilm formation and bacterial colonization could be explained by altered mucin secretion dynamics, the modulation of adherence molecules on the surface of host cells, and increased iron availability in the extracellular medium during coinfections with hRSV [44,52,53]. Moreover, differential gene expression patterns have been observed for *S. aureus*, especially regarding protein and lipid metabolism [44], and it would be interesting to evaluate transcriptomic changes during coinfections for other pathogenic bacteria. Another common feature observed for hRSV–bacteria coinfections is the upregulation of serum CRP [40], which could be interpreted as a dysregulation of inflammatory mechanisms contributing to pathogenesis. This hypothesis is further supported by the enhanced IL-6 secretion in the lung observed for *Moraxella*–hRSV coinfections [64], as well as the modulation of other pro- and anti-inflammatory cytokines [44]. Moreover, enhanced neutrophil infiltration to the lungs and enhanced neutrophil-attracting chemokine CXCL8 are also observed [61,64].

On the other hand, viral coinfections with hRSV tend to inhibit viral replication of either pathogen [104,121]. The response of the infected cell to the virus modulates the expression of intrinsic antiviral factors, such as those of the IFIT and IFI families for IAV [104]. The modulation of other antiviral molecules, such as type I and III interferons (IFN-α, IFN-β, IFN-λ), has also been seen for coinfections with hMPV [121], and IFN-γ secretion in T cells has seen to be impaired during coinfections with IAV [101]. While the modulation of interferons as soluble antiviral molecules and the expression of intrinsic antiviral factors are well studied, there is little information about differences in the immune cell types recruited to the lungs. It has been shown that IAV–hRSV coinfection leads to decreased infiltration of DCs, helper T cells, cytotoxic T cells, and, presumably, NKT cells [108].

We are certain that research on the molecular and cellular mechanisms that mediate hRSV coinfection will provide further insights into hRSV pathogenesis and how it interacts with other microbial agents in our bodies. Undoubtedly, our understanding of hRSV coinfections with both bacteria and viruses is a road yet to be fully paved, which can lead to better treatments of severe pneumonia in children in the future.

## 5. Conclusions

Considering all the epidemiological data available, it is evident that coinfections involving hRSV are prevalent among children under five years old, especially those involving several pathogenic bacteria. As such, the scientific community must address this issue to understand further the complexities underlying these phenomena. All in all, reports of coinfections and superinfections involving hRSV are mainly epidemiological, and studies focusing on molecular and cellular aspects of hRSV–virus and hRSV–bacteria coinfections are scarce. Undoubtedly, understanding the mechanisms that support the high prevalence of these coinfections is tremendously valuable since these can serve as starting points for developing new and more effective treatments and prophylactic approaches that relieve some of the burdens that acute lower respiratory tract infections impose on the health of at-risk patients.

Specifically, mechanisms that explain the exacerbation of pneumonia during hRSV–bacteria coinfections could be discovered if more attention were given to the interaction of the coinfection with the host’s immune system. In this line, experiments involving animal models can shed some light on the complexity of the interaction of these pathogens with the immune system when infecting at or near the same time.

Regarding hRSV–virus coinfections, the hypothesis that one virus restricts the infection of a second virus because of enhanced IFN secretion seems to hold to some extent. This probably contributes to the lower-than-expected rates of coinfection between hRSV and other respiratory viruses. Nonetheless, it is interesting to highlight that hRSV coinfections are amongst the most common for some respiratory viruses, such as hRV and IAV. What are the viral and (or) immune mechanisms that can account for this enhanced coinfection rate? Hopefully, future research will be able to answer this and many other questions regarding the virology and immunology of these infections.

Lastly, it would be interesting to pay attention to the possibly overlooked interaction between hRSV and other non-respiratory pathogens. Do interactions between hRSV and, for example, intestinal pathogens exist? If so, what kind of pathogens are these, and what pathogenic and immune components participate in the interaction between both pathogens, or either pathogen and the immune system of the host? These intriguing questions surely could broaden our view about the extent to which a respiratory infection could modulate homeostasis at a physiological level.

## Data Availability

Not applicable.
